# Low renal but high extrarenal phenotype variability in Schimke immuno-osseous dysplasia

**DOI:** 10.1371/journal.pone.0180926

**Published:** 2017-08-10

**Authors:** Beata S. Lipska-Ziętkiewicz, Jutta Gellermann, Olivia Boyer, Olivier Gribouval, Szymon Ziętkiewicz, Jameela A. Kari, Mohamed A. Shalaby, Fatih Ozaltin, Jiri Dusek, Anette Melk, Aysun K. Bayazit, Laura Massella, Lidia Hyla-Klekot, Sandra Habbig, Astrid Godron, Maria Szczepańska, Beata Bieniaś, Dorota Drożdż, Rasha Odeh, Wioletta Jarmużek, Katarzyna Zachwieja, Agnes Trautmann, Corinne Antignac, Franz Schaefer, on behalf of The PodoNet Consortium

**Affiliations:** 1 Department of Biology and Medical Genetics, Clinical Genetics Unit, Medical University of Gdansk, Gdansk, Poland; 2 Department of Pediatric Nephrology, Charité Universitätsmedizin Berlin, Charité Children's Hospital, Berlin, Germany; 3 Inserm U1163, Imagine Institute, Paris Descartes University, Paris, France; 4 Pediatric Nephrology, Necker Hospital, Assistance Publique - Hôpitaux de Paris, Paris, France; 5 Department of Molecular and Cellular Biology, Intercollegiate Faculty of Biotechnology, University of Gdańsk, Gdańsk, Poland; 6 Pediatric Nephrology Center of Excellence, Pediatrics Department, Faculty of Medicine, King Abdulaziz University, Jeddah, Kingdom of Saudi Arabia; 7 Nephrogenetics Laboratory, Department of Pediatric Nephrology, Hacettepe University Faculty of Medicine, Ankara, Turkey; 8 Department of Pediatric Nephrology, Hacettepe University Faculty of Medicine, Ankara, Turkey; 9 Hacettepe University Center for Biobanking and Genomics, Ankara, Turkey; 10 Department of Pediatrics, University Hospital Motol, Prague, Czech Republic; 11 Pediatric Kidney, Liver and Metabolic Disease, MHH Children´s Hospital, Hannover, Germany; 12 Department of Pediatric Nephrology, Cukurova University, Adana, Turkey; 13 Nephrology and Dialysis Unit, Pediatric Subspecialties Department, Bambino Gesú Children’s Hospital, IRCCS, Rome, Italy; 14 Department of Pediatric Nephrology, Pediatrics and Oncology Center, Chorzów, Poland; 15 Department of Pediatric Nephrology, University Children's Hospital Cologne, Germany; 16 Pediatric Nephrology Unit, Department of Pediatrics, Bordeaux University Hospital, Bordeaux, France; 17 Chair and Department of Pediatrics, SMDZ in Zabrze, Medical University of Silesia in Katowice, Zabrze, Poland; 18 Department of Pediatric Nephrology, Lublin Medical University, Lublin, Poland; 19 Department of Pediatric Nephrology and Hypertension, Dialysis Unit, Jagiellonian University Medical College, Krakow, Poland; 20 Department of Pediatrics, School of Medicine, University of Jordan, Amman, Jordan; 21 Department of Nephrology, Kidney Transplantation and Hypertension, The Children's Memorial Health Institute, Warsaw, Poland; 22 Division of Pediatric Nephrology, Center for Pediatrics and Adolescent Medicine, University of Heidelberg, Heidelberg, Germany; 23 Department of Genetics, Necker Hospital, Assistance Publique - Hôpitaux de Paris, Paris, France; University of Glasgow, UNITED KINGDOM

## Abstract

Schimke immuno-osseous dysplasia (SIOD) is a rare multisystem disorder with early mortality and steroid-resistant nephrotic syndrome (SRNS) progressing to end-stage kidney disease. We hypothesized that next-generation gene panel sequencing may unsurface oligosymptomatic cases of SIOD with potentially milder disease courses. We analyzed the renal and extrarenal phenotypic spectrum and genotype-phenotype associations in 34 patients from 28 families, the largest *SMARCAL1*-associated nephropathy cohort to date. In 11 patients the diagnosis was made unsuspectedly through SRNS gene panel testing. Renal disease first manifested at median age 4.5 yrs, with focal segmental glmerulosclerosis or minimal change nephropathy on biopsy and rapid progression to end-stage kidney disease (ESKD) at median age 8.7 yrs. Whereas patients diagnosed by phenotype more frequently developed severe extrarenal complications (cerebral ischemic events, septicemia) and were more likely to die before age 10 years than patients identified by SRNS-gene panel screening (88 vs. 40%), the subgroups did not differ with respect to age at proteinuria onset and progression to ESKD. Also, 10 of 11 children diagnosed unsuspectedly by Next Generation Sequencing were small at diagnosis and all showed progressive growth failure. Severe phenotypes were usually associated with biallelic truncating mutations and milder phenotypes with biallelic missense mutations. However, no genotype-phenotype correlation was observed for the renal disease course. In conclusion, while short stature is a reliable clue to SIOD in children with SRNS, other systemic features are highly variable. Our findings support routine *SMARCAL1* testing also in non-syndromic SRNS.

## Introduction

Schimke immuno-osseous dysplasia (SIOD; MIM #242900) is an autosomal recessive disorder characterized by the combination of a progressive proteinuric glomerulopathy with spondyloepiphyseal dysplasia, growth retardation, peculiar dysmorphic features (S1 Fig), episodic lymphopenia, defective cellular immunity, and abnormal skin pigmentation consisting of multiple lentigines. Cerebral infarcts, thrombocytopenia and microdontia are observed less frequently [1]. Early death is common and mainly related to opportunistic infections secondary to deficient T cell function. Therapy is largely limited to the prophylaxis and management of the various disease manifestations, such as dialysis and renal transplantation, supplementation of hematopoietic growth factors for neutropenia, orthopedic surgery as required, preventive antiviral therapies and vaccinations, anticoagulation, immunosuppressive therapy for those with autoimmune manifestations, and thyroid hormone supplementation [1].

The disease is caused by biallelic mutations in the *SMARCAL1* gene, encoding the SWI/SNF-related, matrix-associated, actin-dependent regulator of chromatin subfamily A-like protein-1 (SMARCAL-1) from the SWI2/SNF2 family of ATP-dependent chromatin remodeling proteins [2]. Proteins of this family share a conserved helicase ATPase domain comprising an ATP binding site and a catalytic site for ATP hydrolysis, and use energy from DNA-activated ATP hydrolysis for DNA remodeling. SMARCAL1 interacts specifically with branched DNA structures such as replication forks and is thereby critical to the stability of DNA replication [3]. The precise cellular mechanisms how replication fork malfunctioning leads to the specific phenotype of SIOD are still elusive.

SIOD has been classified as extremely rare (orphan) disease with an estimated prevalence of less than 1 per million. We hypothesized that with the advent of comprehensive gene panel screening more cases with less severe, largely renal-limited phenotypes may be detected.

## Material and methods

The PodoNet registry is an international web-based clinical registry (www.podonet.org) for primary SRNS, congenital nephrotic syndrome or persistent subnephrotic proteinuria with likely genetic disease. The registry study protocol and characterisation of the PodoNet cohort were recently published [4].

*SMARCAL1* screening was performed either through direct Sanger sequencing of the entire coding gene sequence and ~20 nucleotides of the adjacent intronic regions (“phenotypic cases”) or using custom targeted amplicon-based multi-gene NGS panel (Multiplicom, Niel, Belgium) for FSGS and related glomerulopathies (“incidental cases”). High-throughput sequencing was performed using the MiSeq/ HiSeq platform (Illumina, San Diego, CA, USA). All NGS findings were verified by Sanger sequencing, which was also used to test eligible family members. For compound heterozygotes, the presence of mutation in *trans* was confirmed through parental segregation.

The analysis of structural information for *SMARCAL1* protein was performed using SwissModel homology modeling server [5] (https://swissmodel.expasy.org/repository/uniprot/Q9NZC9, last accessed January 3^rd^, 2017), S1 File. The *in-silico* assessment of the effects of the detected novel variants on protein structure and function was based on several online prediction tools, namely PolyPhen2 (http://genetics.bwh.harvard.edu/pph2/), SIFT (PROVEAN) (http://sift.jcvi.org/) and Mutation Taster (http://www.mutationtaster.org/). The effect of the detected mutations on pre-mRNA splicing was performed using Human Splicing Finder 3.0 (http://www.umd.be/HSF3/) to estimate possible alterations in either exonic splicing enhancer or the splice site acceptor/donor motifs.

Detailed clinical information on renal and extrarenal symptoms was obtained on all SIOD patients by way of a standardized questionnaire. For comparison, detailed clinical information from patient cohort with SRNS due to *NPHS2*-associated glomerulopathy (n = 156) was extracted from the PodoNet Registry [4].

Frequencies were compared using chi-squared tests with continuity corrections or Fisher exact test with Freeman-Halton extension for 2x3 tables when applicable. For continuous variables, differences between groups were evaluated using Kruskal-Wallis test for global and Mann-Whitney U tests for pairwise comparisons. Patient and renal survival rates were calculated using Kaplan Meier lifetable analysis. Due to the exploratory character of the analysis, no adjustment for multiple comparisons was done. Statistical analyses were performed using STATISTICA 12 (StatSoft; Tulsa, OK, USA) and SAS 9.4 (SAS Institute; Cary, NC, USA) data analysis software systems.

All patients and/or their legal guardians (if applicable) gave written informed consent and the study was approved by the local ethics committees.

## Results

### Study cohort

1105 cases of steroid resistant nephrotic syndrome (SRNS) from pediatric nephrology clinical centers in European and Middle East countries enrolled through the PodoNet SRNS Registry [4], and the in-house biobanks at Necker Hospital in Paris, France, Medical University of Gdańsk, Poland and Hacettepe University Nephrogenetics Laboratory, Ankara, Turkey were systematically screened as part of SRNS-related gene panel analysis, including 308 patients negative for mutations in the first-line SRNS-associated genes (*NPHS2*, exons 8–9 of *WT1*). *SMARCAL1* mutations were found in 9 patients (0.8%) from mostly consanguineous families. In two families the mutation was found in a further affected sibling. The observed prevalence is identical to the one provided in a recent review of 1783 SRNS families who underwent genetic screening [6].

Moreover, information on 23 patients diagnosed with SIOD based on phenotype evaluation followed by Sanger testing was included in the analysis, yielding a cohort of 34 patients. *SMARCAL1*-positive patients did not cluster to any specific region, country or era. Six patients were previously published; updated long-term follow-up data were obtained from these patients [7–11].

### *SMARCAL1* mutation screening

**Fig 1:**
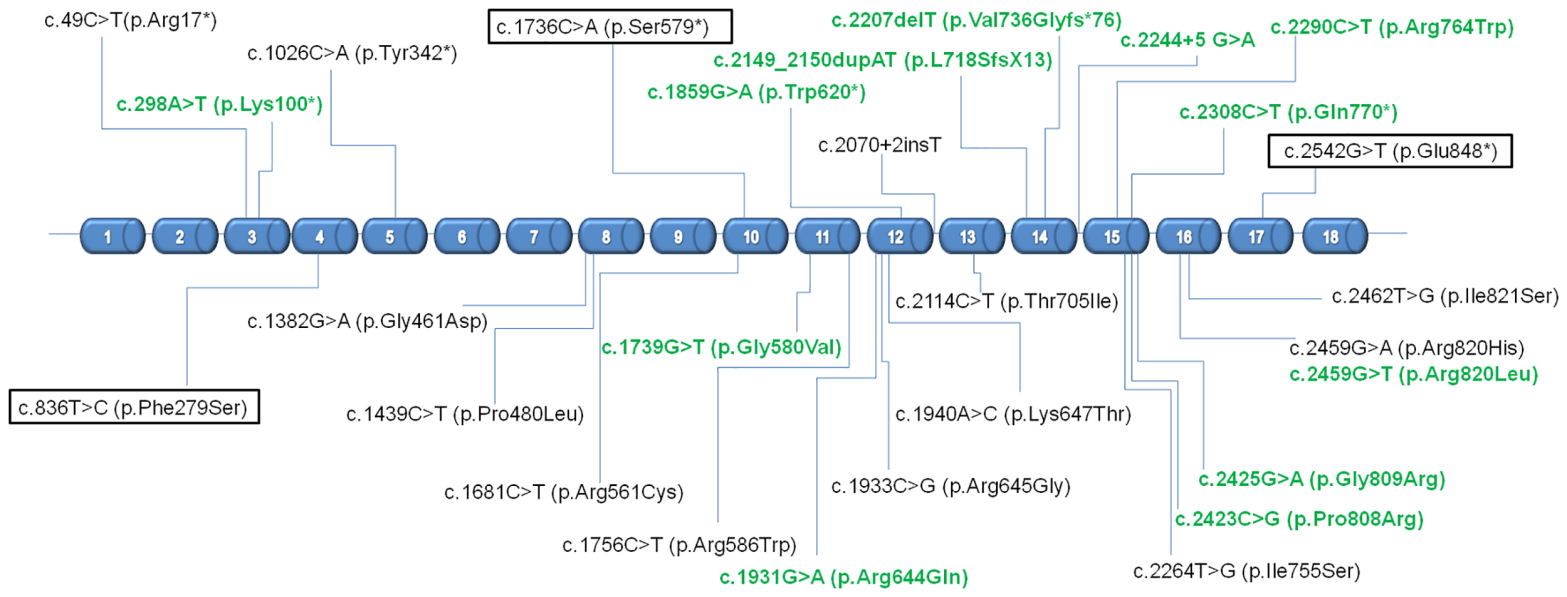
Localization of *SMARCAL1* mutations detected in 34 subjects with Schimke immuno-osseous dysplasia. Legend: $\begin{array}{c} \text{Tagged (black frame)} \end{array}$: recurrent mutations detected in at least three unrelated patients; Green font: novel mutations described for the first time in the current study. Reference sequence: ENST00000357276; NM_014140.

Mutational analysis revealed thirteen novel sequence variants including one large deletion encompassing the entire gene sequence and fifteen previously reported mutations (Fig 1), with half of patients being compound heterozygous. Most of the mutations were located in the helicase C-terminal subdomain, with a hot-spot region in exon 16 (15 patients had at least one mutation there). The analysis of structural information for *SMARCAL1* protein along with *in-silico* assessment of the effects of the mutations on protein structure and function using several online prediction tools classified novel variants as highly likely to be pathogenic. Detailed bioinformatic information on the variants is given in S1 File.

The most common mutation in the cohort was c.2542G>T (p.Glu848*), present in ten patients from eight families of Central-European descent (Polish, Czech, German). This variant has previously been reported exclusively in individuals of European descent with an allele frequency of 0.02% [12].

### Initial manifestation and clinical course of disease

The phenotypic profile is summarized in Table 1, for details refer to S1 Table. Renal disease manifested at median age 4.5 (range: 1.4–17.2) years. 69% of patients presented with nephrotic range proteinuria. Advanced chronic kidney disease (CKD stage 4–5) was present in only two individuals at the time of diagnosis. The two affected siblings identified by family screening exhibited asymptomatic proteinuria. Microscopic hematuria was present in only one patient at disease onset. Focal segmental glomerulosclerosis (FSGS) was found in 82% of biopsies, including three differentiated as collapsing and one as hilar lesion subtypes. The remaining cases were classified as minimal change disease (n = 5). Immunosuppressive therapy was initially applied in 26 out of 33 cases with available treatment information (all unsuspectedly diagnosed and 15 of 21 cases with syndromic phenotype). All patients were resistant to oral steroid therapy. In eight patients initial transient partial responsiveness to calcineurin inhibitors was documented, however the disease inevitably progressed to ESRD within 6 years from diagnosis (median age at ESKD 8.7 (IQR 5.6–10.0) yrs).

**Table 1:** Phenotypic features of patients with Schimke immuno-osseous dysplasia (n = 34).

	Entire cohort	Patients diagnosed by phenotype	Patients diagnosed by genetic screening^a^
N (females)	34 (14)	23 (6)	11 (8)*
Genotype			
Bi-allelic truncating mutation^b^	32.4% (11/34)	47.8% (11/23)	0*
Bi-allelic missense mutation	35.3% (12/34)	21.7% (5/23)	63.6% (7/11)*
Other	35.3% (11/34)	30.4% (7/23)	36.4% (4/11)
Median age at diagnosis (IQR) [years]	4.5 (3.2–7.2)	4.1 (3.0–6.8)	5.7 (4.8–8.4)
Nephrotic range proteinuria at diagnosis	69.0%	66.7%	72.7%
Histopathological findings			
FSGS	81.5% (22/27)	87.5% (14/16)	72.7% (8/11)
MCN	18.5% (5/27)	12.5% (2/16)	27.3% (3/11)
Median age at ESKD (IQR) [years]	8.7 (5.6–10.0)	8.9 (5.6–11.8)	8.7 (6.1–8.9)
Patient survival at age 10 yrs	53.6 ±9.7%	40.4 ± 11.0%	87.5 ± 11.7%*
Intrauterine growth retardation	96.4% (27/28)	100% (20/20)	87.5% (7/8)
Preterm delivery	60.7% (17/28)	70.0% (14/20)	37.5% (3/8)
Height SDS at diagnosis	-3.30 ± 1.46	-3.40 ± 1.46	-3.22 ± 1.50
Height SDS at last observation	-5.24 ± 1.84	-5.81 ± 1.76	-3.81 ± 1.18 **
Lymphocyte abnormalities	83.3% (25/30)	82.6% (19/23)	85.7% (6/7)
Recurrent and/or severe infections	54.8% (17/31)	75.0% (15/20)	18.2% (2/11)*
Abnormal thyroid function	50.0% (15/30)	52.4% (11/21)	44.4% (4/9)
Cerebral ischemic events	39.4% (13/33)	45.5% (10/22)	27.3% (3/11)
Seizures	32.4% (11/34)	34.8% (8/23)	27.3% (3/11)
Cognitive impairment, developmental delay	26.5% (9/34)	34.8% (8/23)	9.1% (1/11)
Lymphoproliferative disorder	8.8% (3/34)	13.6% (3/22)	0
Autoimmune disorders	8.8% (3/34)	8.7% (2/23)	9.1% (1/11)

^a^9 SRNS patients detected incidentally to harbor *SMARCAL1* biallelic mutation through multigenic mutational screening and 2 additional cases of siblings from two families subsequently identified through family testing.

^b^Nonsense, frame-shift, splice-site, large deletion (of the entire gene).

*p<0.05,

**p<0.005.

Numbers in brackets indicate number of patients with feature out of those for whom information was provided.

IQR: interquartile range.

**Fig 2:**
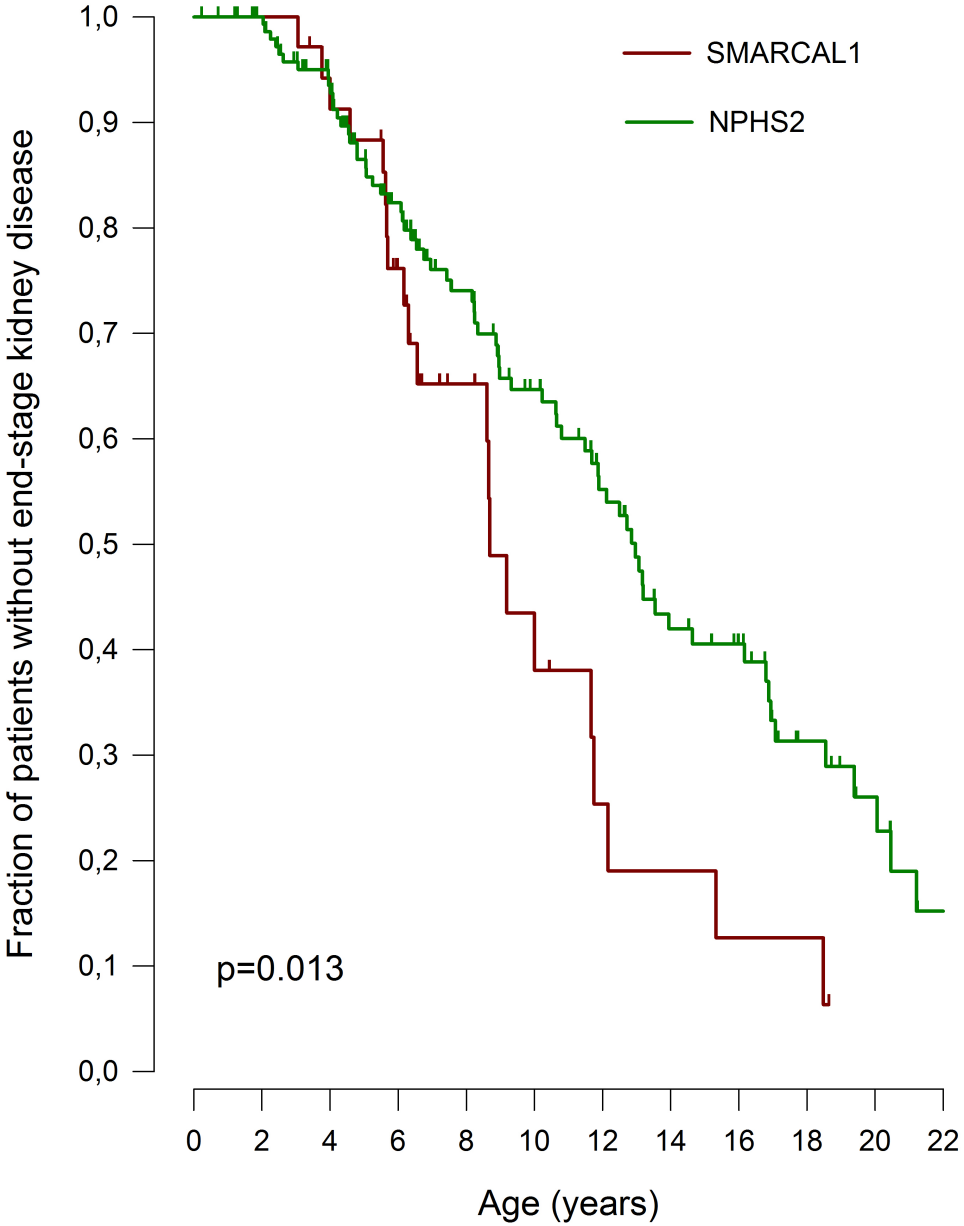
Age at attainment of ESKD in 34 patients with *SMARCAL1* glomerulopathy vs. 156 cases of *NPHS2*-associated SRNS from PodoNet Registry [4].

Renal failure progressed more rapidly than in patients with podocin nephropathy, the most common hereditary podocytopathy. Median ESKD-free survival time was 8.7 (95% CI 8.0–9.4) years for *SMARCAL1* vs. 13.0 (95%CI 11.8–14.1) years for *NPHS2* nephropathy (p = 0.013) (Fig 2).

**Fig 3:**
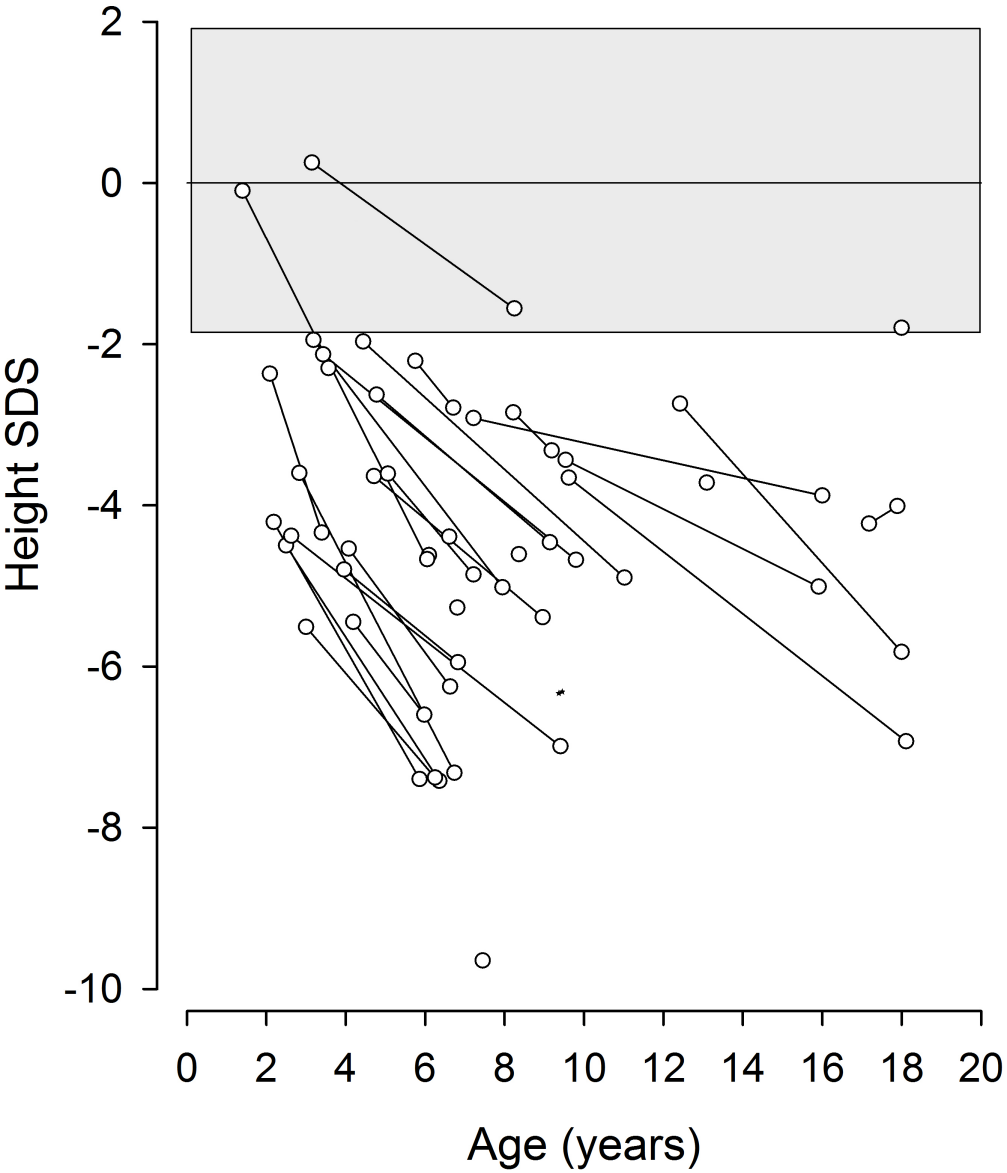
Progressive growth failure in children with Schimke immuno-osseous dysplasia.

Short stature was present in 31/34 patients at time of diagnosis and growth failure was universally observed during a mean follow-up time of 4.7 years (Fig 3). Growth retardation had already been evident *in utero* in 96%, and 61% were born prematurely. Growth hormone therapy, administered in 7 children, was largely ineffective.

83% of patients had hematological abnormalities, the most common being low T cell counts. Two patients developed post-transplant lymphoproliferative disease, two ITP (immune thrombocytopenic purpura), one patient Evans syndrome, and one acute disseminated encephalomyelitis. Eleven patients had recurrent respiratory infections, and six developed severe infections (sepsis, meningitis). 50% of patients had mild to moderate hypothyroidism. Six patients had restrictive lung disease necessitating oxygen therapy. Cerebral ischemic events were reported in 13 (39%) patients with subsequent epilepsy in eleven. Six patients (18%) had moderate cognitive impairment and another three presented with mild developmental delay.

Overall patient survival rate at age 10 years was 54±10%. Six patients died while on conservative treatment, two on dialysis and four post-transplant. The most common causes of death were severe infections (n = 7) and cerebral events (n = 4).

### Characteristics of patients diagnosed incidentally through NGS screening

**Fig 4:**
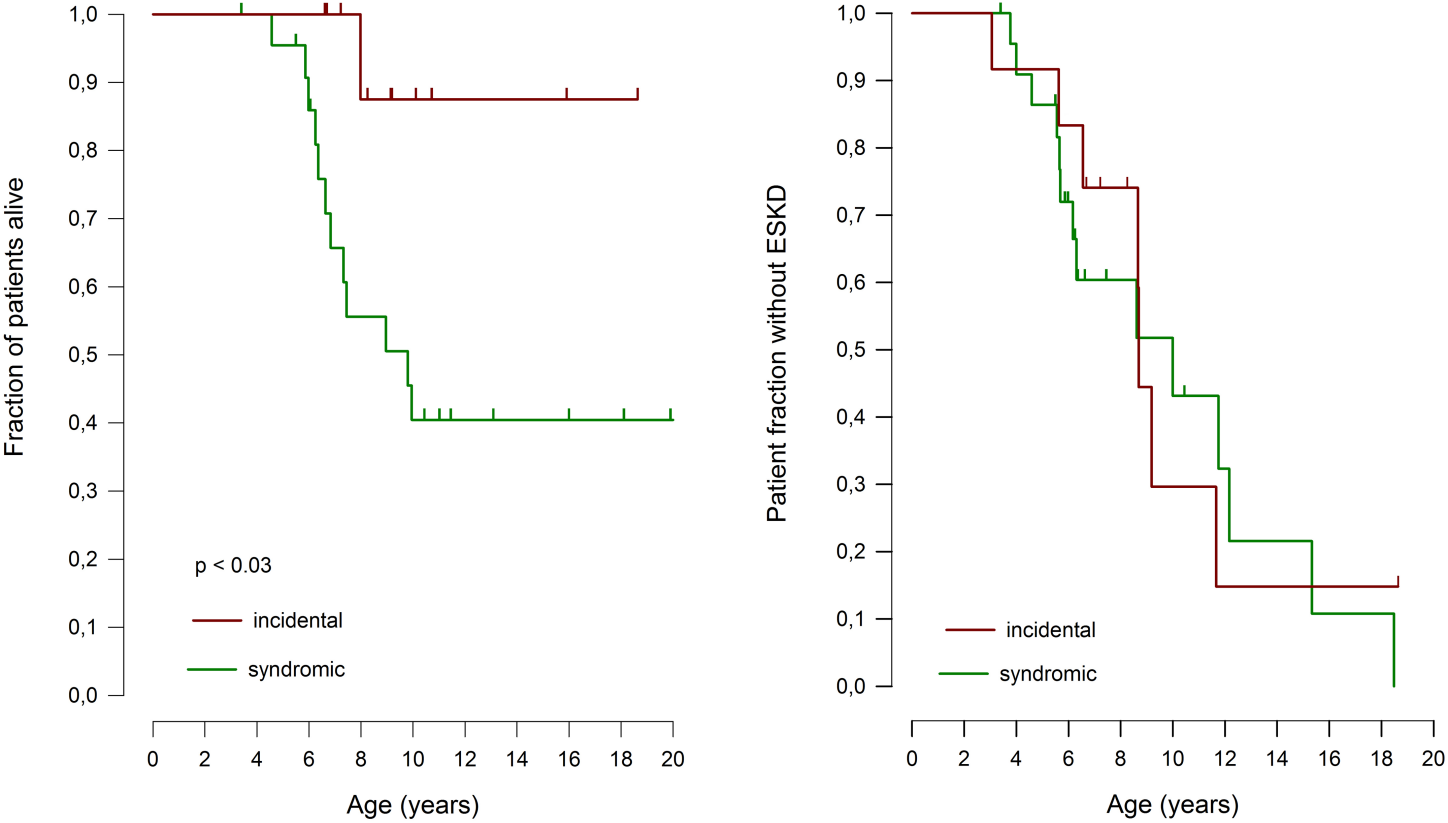
Patient survival (left) and ESKD-free survival rate (right) of patients with Schimke immunoosseous dysplasia diagnosed based on phenotype vs. patients diagnosed incidentally through SRNS-panel gene testing. Patients who deceased before reaching ESKD were censored in the renal survival analysis.

The patients diagnosed unexpectedly by NGS screening showed a milder extrarenal phenotype during follow-up (Table 1), including less frequent infections and no episodes of sepsis, a tendency for less common and less severe cerebral ischemic events, and almost 50% better 10-year patient survival compared to the patients in whom the diagnosis was prompted by the presence of syndromic features (Fig 4). In contrast to their milder extrarenal symptoms the incidentally diagnosed children did not differ from those diagnosed by phenotype with regards to the timing of onset of proteinuria and the rate of progression to ESKD (Fig 4). Importantly in this context, 10 of the 11 incidentally diagnosed patients were small for age when SRNS was first diagnosed and renal function was still normal.

### Genotype-phenotype correlation analysis

Genotype-phenotype correlation analysis of our cohort revealed that 11 patients with bi-allelic truncating (nonsense, frameshift, splice-site or entire gene deletion) mutations exhibited the most severe, and 12 patients with bi-allelic missense mutations the most benign phenotype. Exceptions to this rule were the C-terminal mutations c.2244+5G>A, c.2207delT, and c.2542G>T (p.Glu848*), which were associated with a relatively mild phenotype limited to dysmorphy, skeletal features and renal disease. Patients with bi-allelic truncating mutations exhibited higher mortality than children with bi-allelic missense variants, with 25±15% vs. 67±16% 10-year survival respectively (p = 0.04, S2 Fig). The renal phenotypes did not differ between these groups of genotypes with otherwise high and low respective risk for a severe phenotype, with a similar median age at attainment of ESRD 8.6±1.4 vs. 8.7±1.6 years; p = n.s, S2 Fig).

## Discussion

*SMARCAL1*, the gene defective in SIOD, plays an important role in DNA stabilization and its deficiency leads to impairment of cellular functions due to progressive DNA damage. This results in a progressive systemic disease with early mortality. However, the extent of clinical symptoms and rate of functional deterioration vary to some degree, with some patients manifesting in later childhood and surviving into adulthood [2,13]. Previous reports analyzed cohorts of SIOD patients diagnosed based on their syndromic phenotype, and did not assess the evolution of renal function in detail [1,2,11,13–16]. In this study, we started out from a registry of childhood-onset steroid resistant nephrotic syndrome incorporating SIOD patients diagnosed either on clinical grounds or by comprehensive gene panel screening. We observed a wide and highly variable spectrum of extrarenal symptoms, most of which only emerged over time. Age at diagnosis ranged from 1 to 17 years, and the diagnosis was made unexpectedly by SRNS-gene panel screening in one third of cases. Ninety percent of the incidentally diagnosed children survived to adolescent and adult age, as compared to only 45% of those diagnosed by the classical clinical phenotype. The only invariable extrarenal disease manifestation was short stature, in almost all cases manifesting already at birth with smallness for gestational age and progressing in severity over time. Although growth failure was slightly less severe in the subgroup diagnosed unexpectedly by NGS screening, this observation allows the important clinical conclusion that small stature at the time of diagnosis in a child with otherwise isolated SRNS, in particular when combined with a history of intrauterine growth retardation, is a strong diagnostic clue to SIOD.

Disease severity in SIOD appears inversely proportionate to overall residual *SMARCAL1* activity [15], although substantial phenotypic variation was noted among patients carrying the same mutation even within families [1,16]. Lou *et al*. proposed that infantile-onset, early lethal SIOD would result from presence of at least one *SMARCAL1 null* allele (deletion, nonsense, or frameshift mutation), whereas a juvenile form with less severe phenotype and survival into the second and third decade of life would arise from missense mutations [13,15]. Molecular work has demonstrated that truncating mutations indeed usually cause a loss of SMARCAL1 mRNA and protein, whereas missense mutations alter protein stability, subcellular localisation, chromatin binding and enzymatic activity [15]. In the cohort presented here bi-allelic truncating mutations indeed exhibited a more severe clinical phenotype than patients with bi-allelic missense variants, with a more than twofold difference in 10-year patient survival. However, approximately 50% of SIOD cases are compound heterozygous; in these families genotype-phenotype correlations are not as straightforward. Adding further complexity, we observed that truncating alleles affecting only the C-terminal fragment of the protein appear to associate with a less severe phenotype whereas all but one missense mutations affecting the crucial N-terminal helicase ATP-ase catalytic subdomain caused a clear SIOD phenotype. Hence, the intragenic localization of the lesion needs to be taken into account when inferring from the genotype to the likely phenotype.

SMARCAL1 is expressed in all renal cell lineages during kidney development, and in some but not all glomerular endothelial cells and podocytes, tubular epithelial and collecting duct cells of the mature human kidney [14]. Renal cells of SIOD patients show increased DNA fragmentation [14]. Remarkably, despite ubiquitous expression of the defective gene from earliest stages of kidney development the renal phenotype is initially limited to a proteinuric glomerulopathy manifesting years after birth, usually with FSGS or even minimal change disease histopathology and normal GFR at time of diagnosis in the vast majority of patients.

The diagnosis of asymptomatic proteinuria in two affected siblings detected by family screening suggests that glomerular dysfunction probably develops gradually over time. While relatively slow in onset, once manifest the nephropathy inevitably progresses to end-stage kidney disease. We observed the progression of renal failure in SIOD to be more rapid than in children with podocin nephropathy, the most common form of recessive hereditary SRNS. We speculate that the more aggressive course of renal failure in patients with *SMARCAL1*-associated disease relative to podocin deficiency—which exclusively affects the podocyte—may be related to additional functional effects of defective SMARCAL1 expressed in other nephron structures.

Most notably, in striking contrast to the extrarenal SIOD phenotype neither the onset nor the course of renal disease exhibited any correlation with the type of genetic lesion. Patients with bi-allelic missense mutations did not differ in renal survival from those with bi-allelic truncating mutations. Likewise, the renal prognosis was equally poor in patients diagnosed incidentally by gene panel screening and in those who were diagnosed on clinical grounds by their syndromic phenotype. This unexpected finding indicates a high sensitivity of the renal tissues to the cumulative effects of genomic instability. We speculate that the podocyte, as a post-mitotic cell type, may be particularly vulnerable to this pathogenic mechanism since dysfunctional cells cannot be replaced by proliferation of less affected cells. As a consequence, even minor genetic lesions with retained residual enzymatic activity would lead to progressive loss of the glomerular filtration barrier and progressive proteinuria-related renal damage.

## Conclusions

In conclusion, our study demonstrates that a considerable fraction of patients with SRNS due to SIOD is oligosymptomatic, presenting only with proteinuria and short stature. Whereas extrarenal symptoms and patient survival vary widely and correlate with the type of genetic abnormality, the proteinuric glomerulopathy invariably progresses to end-stage kidney disease within the first two decades of life. It can be expected that the detection rate of milder SIOD variants will increase with routine NGS gene panel screening. *SMARCAL1* should be included in SRNS/FSGS gene panels.

## Supporting information

S1 Fig Phenotype of a patient with Schimke immunoosseous dysplasia. (PDF)

S2 Fig Patient survival and ESKD-free survival rate of patients with Schimke immunoosseous dysplasia diagnosed with bi-allelic truncating mutations vs. patients diagnosed with bi-allelic missense mutations. (PDF)

S1 Table Clinical characteristics of the 34 patients with bi-allelic mutations in the *SMARCAL1* gene. (PDF)

S1 File Pathogenicity assessment of the detected novel sequence variants in *SMARCAL1*. *A*. *In-silico analyses of variant effects on protein structure and function*Summary of bioinformatic analyses of the detected novel sequence variants in the *SMARCAL1* gene.*B*. *Homology-Based Structure Modelling of SMARCAL1 Variant Effects*Structural characteristics of the SMARCAL1 protein.
(PDF)
