# Supplementary material for: Low renal but high extrarenal phenotype variability in Schimke immuno-osseous dysplasia
Source: PLoS One. 2017 Aug 10;12(8):e0180926. doi: 10.1371/journal.pone.0180926 (PMC5552097; doi:10.1371/journal.pone.0180926)

**S1 Figure. Phenotype of a patient with Schimke immunoosseous dysplasia.**

Note characteristic facial features: wide, depressed nasal bridge and a broad nasal tip; short neck and trunk with lumbar lordosis and a protruding abdomen with hyperpigmented macules.

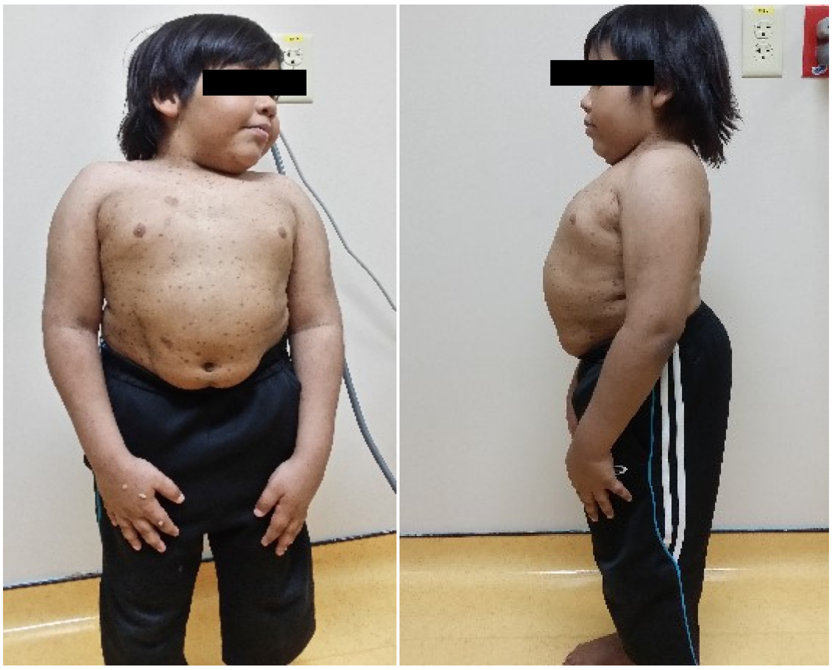

Supplement: S1 Fig — (PDF) [file pone.0180926.s001.pdf]
