# Supplementary material for: Low renal but high extrarenal phenotype variability in Schimke immuno-osseous dysplasia
Source: PLoS One. 2017 Aug 10;12(8):e0180926. doi: 10.1371/journal.pone.0180926 (PMC5552097; doi:10.1371/journal.pone.0180926)

S-2 FIGURE. Patient survival (left) and ESKD-free survival rate (right) of patients with Schimke immunoosseous dysplasia diagnosed with bi-allelic truncating mutations vs. patients diagnosed with bi-allelic missense mutations. Patients who deceased before reaching ESKD were censored in the renal survival analysis.

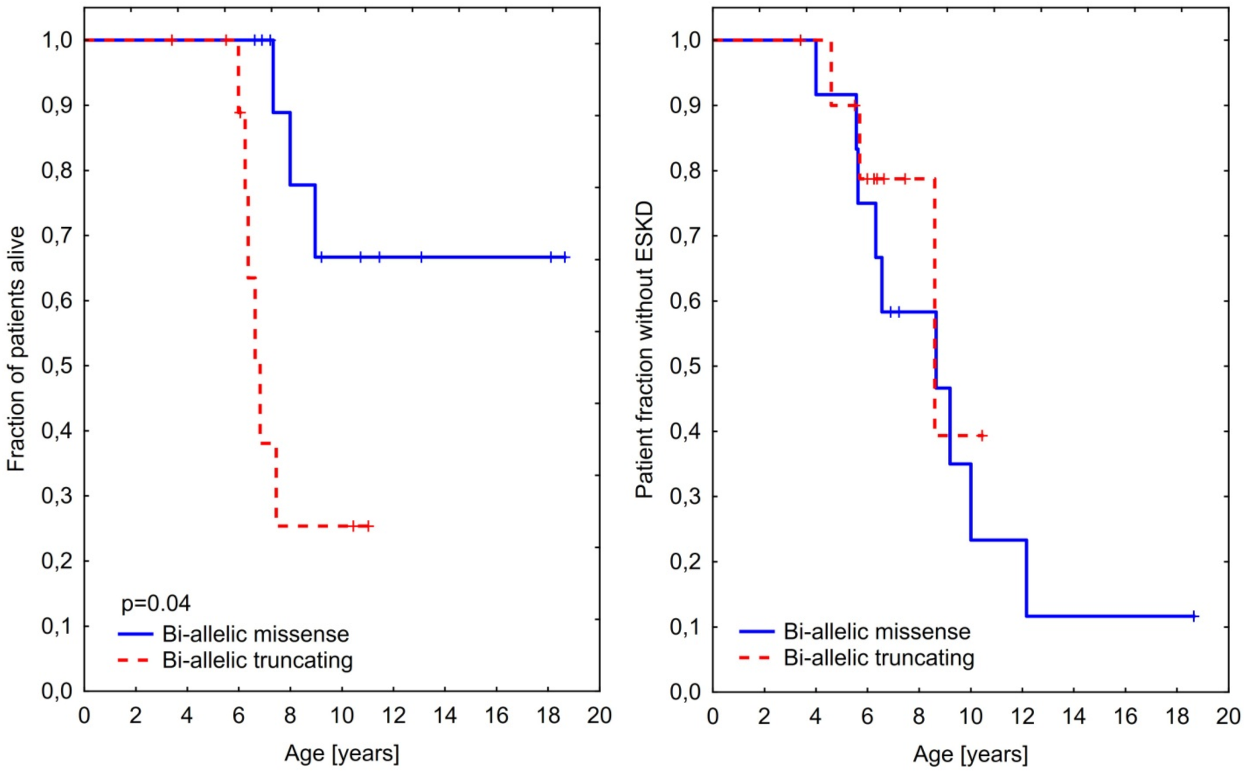

Supplement: S2 Fig — (PDF) [file pone.0180926.s002.pdf]
