# Supplementary material for: Low renal but high extrarenal phenotype variability in Schimke immuno-osseous dysplasia
Source: PLoS One. 2017 Aug 10;12(8):e0180926. doi: 10.1371/journal.pone.0180926 (PMC5552097; doi:10.1371/journal.pone.0180926)
Supplement: S1 Table — (PDF) [file pone.0180926.s003.pdf]

S1 Table. Clinical characteristics of the 34 patients with bi-allelic mutations in the *SMARCAL1* gene.

Family ID (reference if already published)	Mutation(s)	Diagnosis	Gender	Country of origin	Age at 1st manifestation (years)	Initial proteinuria	Initial GFR ml/min/1.73m^2	Histopathology	Age at ESRD (years)	Age at last observation (years)	Response to immunosuppressive treatment	Neurological signs and symptoms	Infections, immunodeficiency, autoimmune diseases ,lympho-proliferative disorders	Other extra-renal signs	Thyroid function	Height at 1st manifestation (SD)	Height at last observation (SD)
1	c.49C>T (p.Arg17*) hom.	phenotype	F	Germany	n/a	0.3g/dl	113	n/a	—	7.45 (death)	n/a	TIAs, migraine, multiple ischemic strokes, secondary focal seizures	—	developmental and speech delay, multiple pigmented nevi, malposition of teeth, IUGR	normal	n/a	-9.7
2	c.49C>T (p.Arg17*)<br>c.836T>C (p.Phe279Ser)	SRNS-gene panel	F	Germany	9.5	14.1 g/g uPCr	93	collapsing FSGS	11.7	15.9	no response to steroids, MMF, *Rituximab*	isolated high-frequency hearing loss	low CD4-helper	neonatal asphyxia resulting in developmental and speech delay, mild/moderate ID, IUGR, preterm delivery	hypo-thyroidism	-3.4	-5.0
3	c.298A>T (p.Lys100*)<br>c.2114C>T (p.Thr705Ile)	phenotype	F	Germany	7.2	4.9g/m^2/day	189	FSGS	11.8	16.0	no response to steroids, CsA, CP,MMF, *Tacrolimus, Rituximab*	—	T-cell deficiency	restrictive lung disease, multiple pigmented nevi, IUGR, preterm delivery	normal	-2.9	-3.9
4-I	c.836T>C (p.Phe279Ser)<br>c.2542G>T (p.Glu848*)	phenotype	M	Germany	13.0	n/a	12	n/a	15.3	34.1	not treated with steroids/CsA/other	—	n/a	—	normal	n/a	-1.8
4-II	c.836T>C (p.Phe279Ser)<br>c.2542G>T (p.Glu848*)	phenotype	M	Germany	12.4	2 g/day	75	FSGS	18.5	31.2	not treated with steroids/CsA/other	TIAs, multiple ischemic strokes, secondary seizures	lymphopenia, low CD4, CD8	restrictive lung disease, IUGR, preterm delivery<br>factor V (Leiden) mutation	hypo-thyroidism	-2.7	-5.8
5-I	c.836T>C (p.Phe279Ser)<br>c.2542G>T (p.Glu848*)	SRNS-gene panel	F	Poland	4.8	3.2 g/day	119	minimal change disease	8.7	9.1	no response to steroids<br>transient response to CsA	TIAs, migraine	frequent respiratory infections;<br>low CD4-helper	Painful muscle contractions and creatine kinase serum levels >2,000 IU/l during infection; IUGR, preterm delivery	latent hypo-thyroidism	-2.6	-4.5
6 (ref.5;13)	c.1026C>A (p.Tyr342*)<br>c.2264T>G (p.Ile755Ser)	phenotype	M	Italy	3.6	n/a	47	collapsing FSGS	3.8	4.6 (death)	no response to steroids/CsA	TIAs, secondary seizures, lower extremity hypotonia	sepsis, persistent diarrhea,<br>lymphopenia, low CD4, CD8	moderate ID, microdontia, agenesia of the 5th tooth (lower arch), solitary kidney, multiple pigmented nevi, IUGR, preterm delivery	n/a	-2.3	n/a

7	c.1382G>A (p.Gly461Asp) hom.	phenotype	M	Czech	4.1	16 g/day	153	FSGS/GGS	5.6	11.5	not treated with steroids/CsA/other	neonatal hypotonia	frequent respiratory infections<br>low CD4, CD8	mild developmental delay, malformed deciduous molars, multiple pigmented nevi, IUGR	normal	-1.9	n/a
8 (ref.9)	c.1439C>T (p.Pro480Leu)<br>c.2264T>G (p.Ile755Ser)	phenotype	F	Italy	4.7	n/a	107	n/a	6.3	9.0 (death)	no response to steroids, CsA	–	catheter-related sepsis, pulmonary Aspergillosis ITP lymphopenia, low IgG	restrictive lung disease, developmental delay,mild ID, microdontia, IUGR, preterm delivery	hypo-thyroidism	-3.6	-5.4
9 (ref.6)	c.1439C>T (p.Pro480Leu)<br>c.2542G>T (p.Glu848*)	phenotype	F	Czech	2.2	1-2 g/m^2/d	114	FSGS/GGS	–	5.7 (death)	not treated with steroids/CsA/other	TIAs, multiple ischemic strokes, secondary seizures, triplegia with motoric aphasia	fungal sepsis<br>self-limited neonatal thrombocytopenia; Evans syndrome (age 5yrs)<br>low CD4, CD8	mild developmental delay, multiple pigmented nevi, IUGR	normal	-4.2	-7.4
10	c.1681C>T (p.Arg561Cys) hom.	SRNS-gene panel	F	Turkey	17.2	2.1 g/day	81	FSGS	–	18.6	no response to steroids, Rituximab<br>partial response to CsA	–	–	–	normal	-4.2	-4.1
11	c.1736C>A (p.Ser579*) hom.	phenotype	M	Lebanon	4.1	28g/g uPCr	59	FSGS	–	6.6 (death)	no response to steroids<br>partial response to CsA	TIAs, multiple ischemic strokes, secondary seizures, hemiparesis	frequent respiratory infections<br>PTLD of the colon (EBV assoc.) non Hodgkin B-cell lymphoma<br>low CD4-helper	mild developmental delay, moderate ID; ASD-II; PFO, dystopia of the right kidney, bilateral hernia inguinalis, IUGR, preterm delivery<br>t-PA deficiency and elevated PAI-I (hom.)	hypo-thyroidism	-4.5	-6.3
12-I	c.1736C>A (p.Ser579*) hom.	phenotype	F	Lebanon	4.0	18.3g/g uPCr	145	FSGS	5.7	6.8 (death)	no response to steroids<br>partial response to CsA, *Tacrolimus*	–	PTLD of the CNS and lung (EBV assoc.) non Hodgkin B-cell lymphoma<br>T-cell deficiency	restrictive lung disease requiring O_2 therapy; ASD, PFO, IUGR, preterm delivery	hypo-thyroidism	-4.8	-6.0
12-II	c.1736C>A (p.Ser579*) hom.	phenotype	M	Lebanon	3.0	3.4g/g uPCr	107	not performed	–	6.3 (death)	no response to steroids<br>partial response to *Tacrolimus*	–	frequent respiratory infections<br>low CD4, CD8	restrictive lung disease requiring O_2 therapy; multiple pigmented nevi, IUGR	hypo-thyroidism	-5.5	-7.4
13	c.1739G>T (p.Gly580Val)<br>c.2423C>G (p.Pro808Arg)	phenotype	M	Macedonia	2.8	+++ (dipstick)	68	not performed	4.0	7.3 (death)	not treated with steroids/CsA/other	periventricular white matter defects, secondary seizures,	frequent respiratory infections<br>mild leukopenia	microdontia, mild developmental delay, no perinatal data	latent hypo-thyroidism	-3.6	-7.3

14	c.1756C>T (p.Arg586Trp) hom.	phenotype	M	Turkey	9.6	9.6g/g uPCr	158	FSGS	10.0	18.1	no response to steroids, CsA, CP	—	meningitis,<br>MDS susp.<br>mild leucopenia, low CD4	post-transplant BK virus asscociated nephropathy (BKVAN); multiple pigmented nevi, IUGR	normal	-3.7	-6.9
15<br>(ref.8)	c1859G>A (p.Trp620*)<br>c.2542G>T (p.Glu848*)	phenotype	M	Poland	4.4	9.1g/day	91	hilar FSGS	8.6	11.2	no response to steroids, CsA<br>partial response to MMF	—	frequent respiratory infections<br>post-transplant ITP<br>mild leucopenia, low CD4, CD8, CD3, IgG	multiple pigmented nevi, IUGR, preterm delivery	normal	-2.0	-4.9
16	c.1931G>A (p.Arg644Gln)<br>c.2542G>T (p.Glu848*)	SRNS-gene panel	M	Poland	3.2	0.16g/day	206	minimal change disease	—	8.3	no response to steroids<br>partial response to CsA	—	mild leucopenia, low CD4	microdontia, delayed permanent teething, multiple pigmented nevi, IUGR	n/a	0.3	-1.6
17	c.1933C>G (p.Arg645Gly)<br>c.2425G>A (p.Gly809Arg)	phenotype	M	France	10.8	n/a	<60	FSGS	12.2	13.1	no response to steroids, CsA, *Tacrolimus*	ischemic stroke, demyelinating peripheral neuropathy, epilepsy (absence)	catheter-related sepsis<br>SCID	femoral head necrosis, multiple pigmented nevi, IUGR, preterm delivery	normal	n/a	-3.7
18	c.1940A>C (p.Lys647Thr) hom.	SRNS-gene panel	F	Morocco	5.7	6.3g/g uPCr	128	minimal change disease	—	6.7	no response to steroids, CsA MMF,	migraine-like headaches possible episode of TIA, epilepsy (absence)	mild leucopenia, low CD4, CD8	severe photophobia, macular opacities, IUGR	normal	-2.2	-2.8
19	c.1940A>C (p.Lys647Thr) hom.	SRNS-gene panel	F	Algeria	5.3	1.4g/day	~60	collapsing FSGS	5.6	6.6	no response to steroids; no CsA/other treatment	—	mild leucopenia, lymphocyte subpopulation tests not performed	chronic vomiting and diarrhea, IUGR	normal	n/a	-4.4
20-I	c.2070+2insT<br>c. 2462T>G (p.Ile821Ser)	phenotype	M	France	3.4	n/a	n/a	FSGS	5.7	9.8 (death)	no response to steroids<br>partial response to CsA	TIAs, secondary seizures	frequent respiratory infections<br>mild leucopenia, lymphopenia, low IgG	restrictive lung disease requiring O_2 therapy during infections; multiple pigmented nevi, malposition of teeth, IUGR, preterm delivery	hypo-thyroidism	-2.1	-4.7
20-II	c.2070+2insT<br>c. 2462T>G (p.Ile821Ser)	phenotype	M	France	3.2	0.7g/l	105	minimal change disease	6.2	8.0 (death at 10 yrs)	no response to steroids<br>partial response to CsA	TIAs, ischemic stroke, secondary seizures, hemiplegia	frequent respiratory infections<br>SCID	hypertrophic and dilated cardiomyopathy with acute and transient heart failure (age 6yrs) multiple pigmented nevi, IUGR, preterm delivery	normal	-2.0	-5.0
21	c.2149_2150dupAT (p.Leu718Serfs*13)<br>c.2542G>T (p.Glu848*)	phenotype	M	Germany	2.1	8.2g/g uPCr	112	minimal change disease	—	3.4	no response to steroids, CsA	—	n/a<br>low CD4	photophobia, multiple pigmented nevi, IUGR, preterm delivery	latent hypo-thyroidism	-2.4	-4.3

22 (ref.7)	c.2207delT (p.Val736Glyfs*75) hom.	phenotype	M	Saudi Arabia (Yamani)	6.8	7.4g/l	129	FSGS	—	10.4	no response to steroids, CsA, CP	—	frequent respiratory infections	loose, fragile, and yellow-stained primary teeth, polyhydramnion, IUGR	hypo-thyroidism	-5.3	n/a
23	c.2244+5 G>A hom.	phenotype	M	United Kingdom	4.5	n/a	n/a	not performed	—	5.5	not treated with steroids/CsA/other	n/a	n/a	mild developmental delay,mild ID, no perinatal data	n/a	n/a	n/a
24-I	c.2290C>T (p.Arg764Trp) hom.	SRNS-gene panel	M	Saudi Arabia	8.4	+++ (dipstick)	28	FSGS	8.7	10.7	no response to steroids, MMF	generalized tonic-clonic seizures secondary to ESKD	lymphocyte subpopulation tests not performed	multiple pigmented nevi, IUGR	hypo-thyroidism	-4.6	n/a
24-II	c.2290C>T (p.Arg764Trp) hom.	family screening	F	Saudi Arabia	5.1	4g/l	180	FSGS	—	7.2	no response to steroids, CsA	—	lymphocyte subpopulation tests not performed	multiple pigmented nevi, IUGR	latent hypo-thyroidism	-3.6	-4.9
25	c.2308C>T (p.Gln770*) hom.	phenotype	M	Jordan	4.2	9.3g/g uPCr	252	FSGS	—	6.0 (death)	no response to steroids, MMF	ischemic stroke, migraine	Sepsis<br>low CD4	multiple pigmented nevi, clinodactyly, syndactyly of toes, IUGR, preterm delivery	latent hypo-thyroidism	-5.5	-6.6
26	c.2542G>T (p.Glu848*)<br>c.2459G>T (p.Arg820Leu)	SRNS-gene panel	F	Poland	2.6	3g/day	40	FSGS	3.1	10.1	no response to steroids, CsA	migraine-like headaches, focal seizures	acute disseminated encephalomyelitis (ADEM)<br>no meningitis<br>mild leucopenia	multiple pigmented nevi, IUGR, preterm delivery	normal	-4.4	-7.0
27-I	c.2459G>A (p.Arg820His) hom.	SRNS-gene panel	M	Turkey	8.2	'nephrotic range'	52	FSGS	9.2	9.2	no response to steroids, CsA	—	lymphocyte subpopulation tests not performed	no perinatal data	normal	-2.9	-3.3
27-II	c.2459G>A (p.Arg820His) hom.	family screening	F	Turkey	6.1	1.1g/g uPCr	200	FSGS/GGS	6.6	8.0 (death)	no response to steroids, CsA	—	frequent respiratory infections<br>lymphocyte subpopulation tests not performed	no perinatal data	n/a	-4.6	n/a
5-II	c.2542G>T (p Glu848*) hom.	phenotype	M	Poland	1.4	++ (dipstick)	n/a	FSGS	4.6	6.1	no response to steroids, CsA	—	frequent respiratory infections<br>low CD4	microdontia multiple pigmented nevi, oligohydramnion, IUGR, preterm delivery	hypo-thyroidism	-0.1	-4.7
28	c.2542G>T (p Glu848*)<br>del(2)(q34-q36)	phenotype	F	Germany	2.5	1.9g/g uPCr	165	not performed	—	6.4 (death)	not treated with steroids/CsA/other	multiple ischemic strokes, secondary focal seizures,	pancytopenia in infancy T-cell (CD4, CD8) deficiency	developmental delay,moderate ID, corneal opacities, microcornea, oligohydramnion, IUGR, preterm delivery	normal	-4.5	-7.4

Legend:

n/a – not available; hom – homozygous; M – male; F – female; uPCr – urinary protein/creatinine ratio; uAlbCr – urinary albumin/creatinine ratio; FSGS – focal segmental glomerulosclerosis; GGS – global glomerulosclerosis; ESRD – end-stage renal disease; CsA – *ciclosporin A*; MMF – *Mycophenolate mofetil*; CP – *Cyclophosphamide;* TIA – transient ischemic attack; CD – a class of glycoproteins (Cluster of Differentiation); tPA – tissue plasminogen activator; PAI-I – plasminogen activator inhibitor-1 (also known as endothelial plasminogen activator inhibitor or serpin E1); PTLD – Post-transplant lymphoproliferative disorder; CNS – central nervous system; EBV – Ebstein-Barr virus; ITP – immune thrombocytopenic purpura; IgG – immunoglobulin G; MDS – myelodysplastic syndrome; SCID – severe combined immunodeficiency; IUGR – intrauterine growth retardation; ID – intellectual disability; ASD – atrial septum defect; PFO – patent foramen ovale; SD – standard deviation;
