# Supplementary material for: Low renal but high extrarenal phenotype variability in Schimke immuno-osseous dysplasia
Source: PLoS One. 2017 Aug 10;12(8):e0180926. doi: 10.1371/journal.pone.0180926 (PMC5552097; doi:10.1371/journal.pone.0180926)

## S1 File. Pathogenicity assessment of the detected novel sequence variants in *SMARCAL1*.

### *A. In-silico analyses of variant effects on protein structure and function*

**Table.** Summary of bioinformatic analyses of the detected novel sequence variants in the *SMARCAL1* gene.

| Variant (rs ID if applicable) | Exon | Protein change | Protein region | Conservation+ | Prevalence in control population [MAF]* | Grantham difference score | Human Splicing Finder 3.0 | PolyPhen2 Proveran predictors |
|---|---|---|---|---|---|---|---|---|
| **c.298A>T** | 3 | p.Lys100* | loss of both HARP domains and both (ATP binding and C-terminal helicase subdomains | low conservation | not reported | n/a | no significant motifs | n/a |
| **c.1739G>T** | 11 | p.Gly580Val | Helicase ATP-binding subdomain | highly conserved, within conserved region | not reported | 109 | activation of an exonic cryptic donor site | deleterious (PolyPhen 1.0; Proveran -8.923) |
| **c.1859G>A** | 12 | p.Trp620* | loss of helicase C-terminal subdomain | highly conserved, within partially conserved region | not reported | n/a | creation of an exonic ESS site | n/a |
| **c.1931G>A** **rs140515579** | 12 | p. Arg644Gln[1] | linker between ATP binding and C-terminal helicase subdomains | highly conserved, within conserved region | 0.0017% (0.02% in Africans) no homozygotes reported | 43 | alteration of an exonic ESE site | deleterious (PolyPhen 1.0; Proveran -3.841) |
| **c.2149_2150dupAT** | 14 | p.Leu718Ser fs*13 | Helicase C-terminal subdomain | highly conserved, within partially conserved region | not reported | n/a | no significant motifs | n/a |
| **c.2207delT** | 14 | p.Val736Gly fs*76 | Helicase C-terminal subdomain | highly conserved, within partially conserved region | not reported | n/a | creation of an exonic ESS site | n/a |
| **c.2244+5 G>A** | intron 14 | p.? | n/a | n/a | not reported | n/a | disruption of the WT donor site | n/a |
| **c.2290C>T** | 15 | p.Arg764Trp[2] | Helicase C-terminal subdomain | highly conserved, within partially conserved region | not reported | 101 | alteration of (ESS/ESE spicing | deleterious (PolyPhen 1.0; Proveran -7.93) |
| **c.2308C>T** | 15 | p.Gln770* | Helicase C-terminal subdomain | highly conserved, within partially conserved region | not reported | n/a | creation of an exonic ESS site | n/a |
| **c.2423C>G** | 15 | p.Pro808Arg | Helicase C-terminal subdomain | highly conserved, within conserved region | not reported | 103 | creation of an exonic ESS site | deleterious (PolyPhen 1.0; Proveran -8.941 |
| **c.2425G>A** | 15 | p.Gly809Arg | Helicase C-terminal subdomain | highly conserved, within conserved region | not reported | 125 | creation of an exonic ESS site | deleterious (PolyPhen 0.998; Proveran -7.481) |
| **c.2459G>T** | 16 | p.Arg820Leu[3] | Helicase C-terminal subdomain | highly conserved, within conserved region | not reported | 102 | no significant motifs | deleterious (PolyPhen 1.0; Proveran -6.944) |
| **del 2q34-q36** | 1-17 | n/a | entire protein | n/a | n/a | n/a | n/a | n/a |

**Legend:**

Reference seq. ENST00000357276; NM_014140; UniProt peptide Q9NZC9; protein domains established according to Swiss-Prot:P19544.

*MAF – minor allele frequency; estimation based on data of 60,706 multiethnic individual genomes catalogued by the Exome Aggregation Consortium (ExAC (accessed 8th November 2016); + – conservation calculated using ClustalW algorithm; n/a – not applicable; ESE – exonic splicing enhancer; ESS – exonic splicing silencer; WT – wild type

[1] *Boerkoel et al.* (*Nat Genet* 2002; 30:215-20) reported a mutation affecting the same residue: c.1930C>T (p.Arg644Trp) in a SIOD patient.
[2] *Boerkoel et al.* (*Nat Genet* 2002; 30:215-20) reported a mutation affecting the same residue: c.2291G>A (p.Arg764Gln) in a SIOD patient.
[3] *Boerkoel et al.* (*Nat Genet* 2002;30:215-20) reported a mutation affecting the same nucleotide but with a different substitution c.2459G>A (p.Arg820His) in a SIOD patient.

*B. Homology-Based Structure Modelling of SMARCAL1 Variant Effects*

The attempt to elucidate the impact of pathogenic missense mutations on SMARCAL1 protein function is hampered by the limited availability of structural data. To date, only the RPA-binding fragment of human SMARCAL1 (residues 5-30) (PDB ID: 4mqv [1]) and the mouse SMARCAL1 protein fragment corresponding to HARP1 domain have been solved [2]. Since all but one missense mutations found in this study are placed within the helicase ATPase domain (Figure 1A), we searched the SwissModel homology modeling repository for structure models of the helicase ATPase region. This revealed two models, both based on templates of ~25% sequence identity.

**Model 1** (Figure 1B) was built using *Sulfolobus solfataricus* SWI2/SNF2 ATPase core. In this model both subdomains wrap around the DNA molecule, the C-terminal subdomain forming a pocket around the ATPase active site of the N-terminal subdomain. Several mutations found in this work are placed in the immediate vicinity of the ATP binding site, including the novel Gly580Val mutation within the conserved motif III. Besides, the Gly461 residue lies within the highly conserved Walker A ATPase module which is crucial to ATP hydrolysis, and the Arg561 residue interacts with the phosphate backbone of DNA in this model within the "IIa" motif that correlates ATP hydrolysis to DNA binding. Lys647 lies in the linker between subdomains, in the vicinity of the "hinge" region and close to the C-terminal subdomain. This localization may indicate involvement in communication between those substructures.
The mutation sites placed in the C-terminal subdomain also affect conserved motifs: among the novel mutations, the Pro808 residue lies in motif Va, Arg764 in motif IVa and Arg820 in motif VI. The two latter residues were found to be mutated by different substitutions [3].
**Model 2,** based on fungal chromatin remodeller protein MtISWI (Figure 1C) differs from model 1 in the relative positions of the subdomains. Compared to model 1, both substructures retain their fold and similar conformation, but the C-terminal subdomain is rotated approx. 180° relative to the N-terminal subdomain (as marked by an arrow in Figure 1C) by a hinge movement within the region 646-656. The ATP binding site of the N-terminal subdomain is no more in contact with the C-terminal one. This difference as seen in template structures was postulated to depict different phases of ATP-driven conformational changes generating the "power stroke" to translocate protein relative to DNA [4]. Assuming this possibility, the position of residues Arg764, Arg820 and Ile821, mutations of which were

described for the first time in this study, indicate their role in interacting with N-terminal ATPase subdomain, as they contact N-terminal subdomain only in this conformational state.

*Description of Methods*

Homology models for SMARCAL1 protein were retrieved from the SWISS-MODEL repository (accessed January 3rd 2017) [5,6]. Model 1 was based on the *Sulfolobus solfataricus* SWI2/SNF2 ATPase core solved by X-ray diffraction to 3A in complex with ds DNA (1z63.pdb) [4]. The resulting model is spanning from residue 429 to 856 of human SMARCAL1 protein at the sequence identity of 26.6%. DNA coordinates were transferred to the model by aligning both structures using DeepView software [7]. The resulting complex was energy-minimized in GROMACS 2016.1 [8], and to check complex stability, subjected to short 5 ns molecular dynamics simulation in explicit water in AMBER99-SB-ILSDN77 force field.
Model 2 was based on the *Thermothelomyces thermophila* chromatin remodeller protein MtISWI (5jxr.pdb, [9]), which has a sequence identity of 25%. This model was superimposed on model 1 for comparison in DeepView. Both models were visualized using VMD software [10].

## *References*

[1] Xie S, Lu Y, Jakoncic J, *et al*. Structure of RPA32 bound to N-terminus od SMARCAL1 redefines the binding interface between RPA32 and its interacting proteins. *FEBS J* 2014; 281:3382-3396
[2] Mason A, Rambo R, Greer B, *et al.* A structure–specific nucleic acid-binding domain conserved among DNA repair proteins. *PNAS* 2014; 111(21):7618-7623.
[3] Elizondo LI, Cho KS, Zhang W, et al. Schimke immuno-osseous dysplasia: SMARCAL1 loss-of-function and phenotypic correlation. J Med Genet 2009;46:49-59.
[4] Dürr H, Körner C, Müller M, *et al.* X-ray structures of the *Sulfolobus solfataricus* SWI2/SNF2 ATPase core and its complex with DNA. *Cell*. 2005; 121:363-73.
[5] Kiefer F, Arnold K, Kunzli M. *et al*. The SWISS-MODEL Repository and associated resources. *Nucleic Acids Res*. 2009; 37: D387-92.
[6] Biasini M, Bienert S, Waterhouse A. *et al.* SWISS-MODEL: modeling protein tertiary and quaternary structure using evolutionary information. *Nucleic Acids Res*. 2014; 42:W252-W258.
[7] Guex N, Peitsch MC. SWISS-MODEL and the Swiss-PdbViewer: An environment for comparative protein modeling. *Electrophoresis* 1997; 18: 2714-2723. http://www.expasy.org/spdbv/
[8] Abraham MJ, Murtola T, Schulz R *et al.* GROMACS: High performance molecular simulations through multi-level parallelism from laptops to supercomputers. *Software X* 2015; 1–2:19–25.
[9] Yan L, Wang L, Tian Y, *et al.* Structure and regulation of the chromatin remodeller ISWI. *Nature* 2016; 540:466-469.
[10] Humphrey W, Dalke A, Schulten K. "VMD - Visual Molecular Dynamics". *J Molec Graphics* 1996; 14:33-38. http://www.ks.uiuc.edu/Research/vmd/

**Figure 1**. Structural characteristics of the SMARCAL1 protein.

**A) Domain organisation of SMARCAL1 protein.** Upper line: positions of residues altered by mutations found in this work (grey, previously described; red, novel mutations). **B) Homology model of SMARCAL1 protein constructed using 1Z63.pdb template (Model 1).** Blue, N-terminal ATP-binding lobe of helicase ATPase domain; yellow, C-terminal lobe of helicase ATPase domain. Hinge region depicted in orange. ATP binding site motifs marked in green: Walker A (motif I), DESH catalytic residues (motif II), motif III. DNA sensor switch (motif IIa) marked in magenta. Residues affected by missense mutations marked in red ball-and-stick representation (novel mutations in brighter red). **C) Homology model of SMARCAL1 protein constructed using 5jxr.pdb template (Model 2), superimposed on Model1 (grayed out) and color-coded as above.** Arrow marks rotation of C-terminal subdomain.

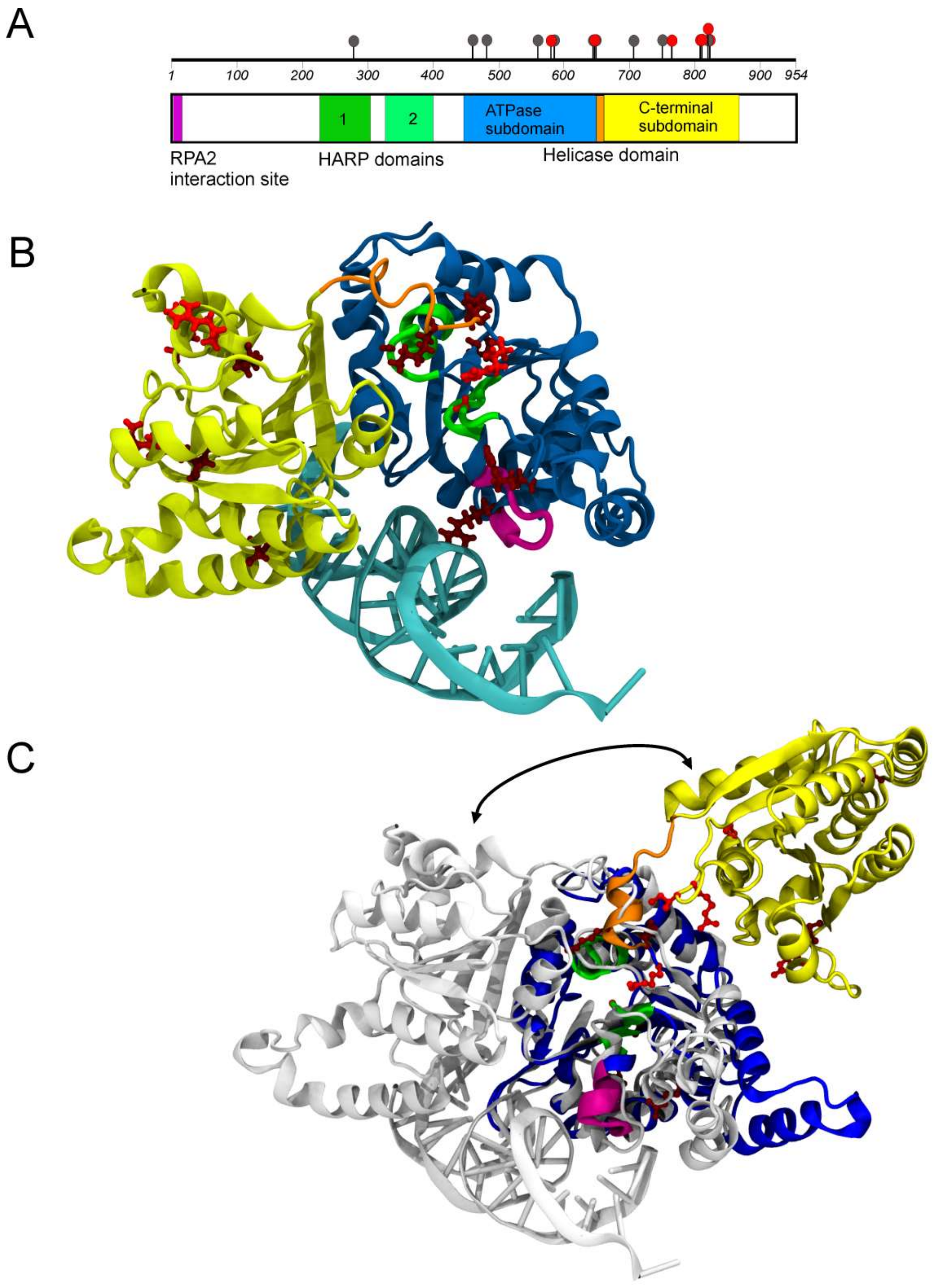

Supplement: S1 File — A. In-silico analyses of variant effects on protein structure and functionSummary of bioinformatic analyses of the detected novel sequence variants in the SMARCAL1 gene.B. Homology-Based Structure Modelling of SMARCAL1 Variant EffectsStructural characteristics of the SMARCAL1 protein. (PDF) [file pone.0180926.s004.pdf]
